# A multidisciplinary approach to rescue a full-term pregnant and her fetus after blunt abdominal trauma: A case report and literature review

**DOI:** 10.1093/jscr/rjac559

**Published:** 2022-12-09

**Authors:** Ismail Mahmood, Husham Abdelrahman, Suhail Hakim, Ayman El-Menyar, Sandro Rizoli, Mohammad Asim, Ammar Al-hassani, Yassir Abdulrahman, Gustav Strandvik, Hassan Al-Thani

**Affiliations:** Department of Surgery, Trauma Surgery, Hamad Medical Corporation, Doha, Qatar; Department of Surgery, Trauma Surgery, Hamad Medical Corporation, Doha, Qatar; Department of Surgery, Trauma Surgery, Hamad Medical Corporation, Doha, Qatar; Department of Surgery, Trauma Surgery, Hamad Medical Corporation, Doha, Qatar; Department of Clinical Medicine, Weill Cornell Medical College, Doha, Qatar; Department of Surgery, Trauma Surgery, Hamad Medical Corporation, Doha, Qatar; Department of Surgery, Trauma Surgery, Hamad Medical Corporation, Doha, Qatar; Department of Surgery, Trauma Surgery, Hamad Medical Corporation, Doha, Qatar; Department of Surgery, Trauma Surgery, Hamad Medical Corporation, Doha, Qatar; Department of Surgery, Trauma Surgery, Hamad Medical Corporation, Doha, Qatar; Department of Surgery, College of Medicine, Qatar University, Doha, Qatar; Department of Surgery, Trauma Surgery, Hamad Medical Corporation, Doha, Qatar

## Abstract

Blunt abdominal trauma due to motor vehicle crash is one of the leading causes of mortality during pregnancy. Though the trauma team plays a critical role in the initial management, a multidisciplinary contribution is essential to ensure the safety of the mother and her fetus. This case report followed the multidisciplinary approach for the management of a 32-year-old female during the last week of pregnancy. She sustained blunt trauma causing maternal and fetal distress due to abruption of the placenta with a large intrauterine and retroplacental hemorrhage, retro-hepatic and retroperitoneal hemorrhage, pseudoaneurysm of uterine arteries leading to postpartum hemorrhage. Immediate intervention and management at a Level 1 trauma center led to survival of both the mother and infant.

## INTRODUCTION

The intensity of pressure due to blunt trauma to the pregnant abdomen can cause placental abruption, preterm delivery, solid organ injury and retroperitoneal hemorrhage. Motor vehicle crash (MVC) is not only the most frequent but also the most life-threatening mechanism of injury. Death rate could be reached 10.7% in the fetuses and 13.7% in the mothers [1, 2]. Fetal morality occurred in 90% of patients who sustained severe injuries (Injury severity score (ISS) > 15) and urgent laparotomy was required in 86% of women presenting with direct trauma to the abdomen [3].

Population-based cohort study of Australian women reported 2147 pregnant women involved in MVC. In 3.3% of patients, the traumatic event led to delivery during the posttrauma hospitalization, 8% had intra-abdominal injuries and seven (10%) were admitted to the ICU, three of whom subsequently died. They concluded that injuries from an MVC during pregnancy are associated with poor outcomes with increased risk of antepartum bleeding, preterm birth, caesarean section and perinatal mortality [4]. Herein, we presented a scenario of how the multidisciplinary approach successfully works to rescue a mother and her fetus post-polytrauma due to MVC.

## CASE REPORT

A 32-year-old G2P1 female who was ~39 weeks pregnant presented to the trauma resuscitation room after being involved in a high-speed motor vehicle crash. She was a restrained front seat passenger who swerved into oncoming traffic and was ‘T-boned’ by a car on her side. She was transported by private car to the Hamad Trauma Center. On arrival, the patient was very stressed and anxious. Initial blood pressure was 110/62 mm Hg, heart rate 98 beats per minute, respiratory rate 18 per minute and temperature 36°C. Primary survey revealed an intact airway with bilateral breath sounds. The patient was alert with a Glasgow Coma Scale (GCS) score of 15.

The nursing staff obtained intravenous (IV) access and completed the relevant diagnostic lab work. O2 supplementation, IV fluids, left lateral tilt were done, and an urgent call-over was given to the on-duty obstetrics (OB) and pediatrics team. The patient had a bruise over her abdomen, which was tender all over. There was no chest wall or spinal tenderness, and her extremities were warm and well perfused with no gross deformities apart from tenderness and swelling at the right shoulder. Per vaginal examination revealed 3-cm cervical dilatation with fresh bleeding.

Focused assessment with sonography in trauma (FAST scan) detected fluid in the right upper abdominal quadrant and E-FAST of the chest was normal. Fetal heartbeats were detected with difficulty, but there was no fetal movement appreciated.

Laboratory tests revealed mild anemia (hemoglobin, 9.8 g/dl; hematocrit, 30%), normal coagulation and mild acidosis (pH 7.3; bicarbonate, 18 mEq/l; lactate, 3.1 mmol/l, base excess −5). Through level one transfuser, O negative blood was transfused, and the blood bank was notified for massive transfusion protocol activation. The patient was immediately transferred to the operating room (OR) without imaging at 12:45 and had an emergency C-section as well as exploratory laparotomy for the evaluation of internal bleeding. The trauma team proceeded with the exploratory laparotomy and after initial midline incision, the OB team performed an emergency C-section. A lower segment incision in the uterus and a female fetus, cyanotic fully covered with blood, weighing 3300 g, was delivered. Abruption of the placenta with large intrauterine and retroplacental clotted and fresh blood was noted (~2 l).

The patient went into cardiac arrest; cardiopulmonary resuscitation (CPR) was initiated, and manual aortic compression was performed. After 1–2 min, the return of spontaneous circulation was achieved, and the patient received red blood cells (RBC) transfusion that stabilized her vitals along with inotropes infusion. The placenta was removed, bleeding was controlled and the uterus was closed by the OB team. Further examination of the abdominal cavity revealed bleeding from right retro-hepatic area which was profuse and continuous. Neither compression nor packing successfully controlled the bleeding, and therefore, the skin incision was extended into an inverted L-shape (right lateral subcostal extension of midline incision) to adequately expose the whole liver.

There was active bleeding from laceration at upper pole right kidney, branches of right adrenal vessels and inferior phrenic veins, which were controlled by direct suturing and ligation. In addition, there was laceration at the posterior surface of the right lobe of the liver (segment 8) with active bleeding. Topical hemostatic agent (Powder, surgical and quick clot) and perihepatic packing were used to achieve hemostasis. The decision was made to leave four sponges packed around the liver and keep the abdomen open for a second look.

**Figure 1:**
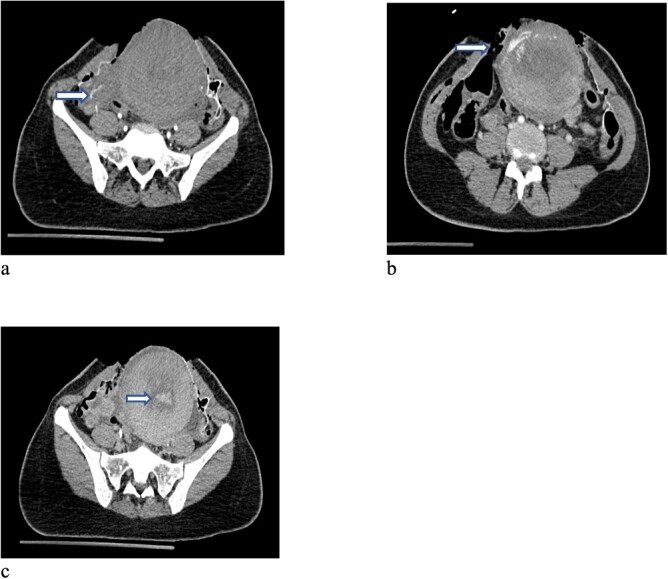
CT scan of the pelvis (**a-c**) after cesarean section revealed focus of contrast blush seen in the arterial phase at the right lateral side of the uterine wall, which increased in the venous and delayed phases suggesting active arterial bleeding. The contrast extravasation is also seen within the endometrial cavity.

**Figure 2:**
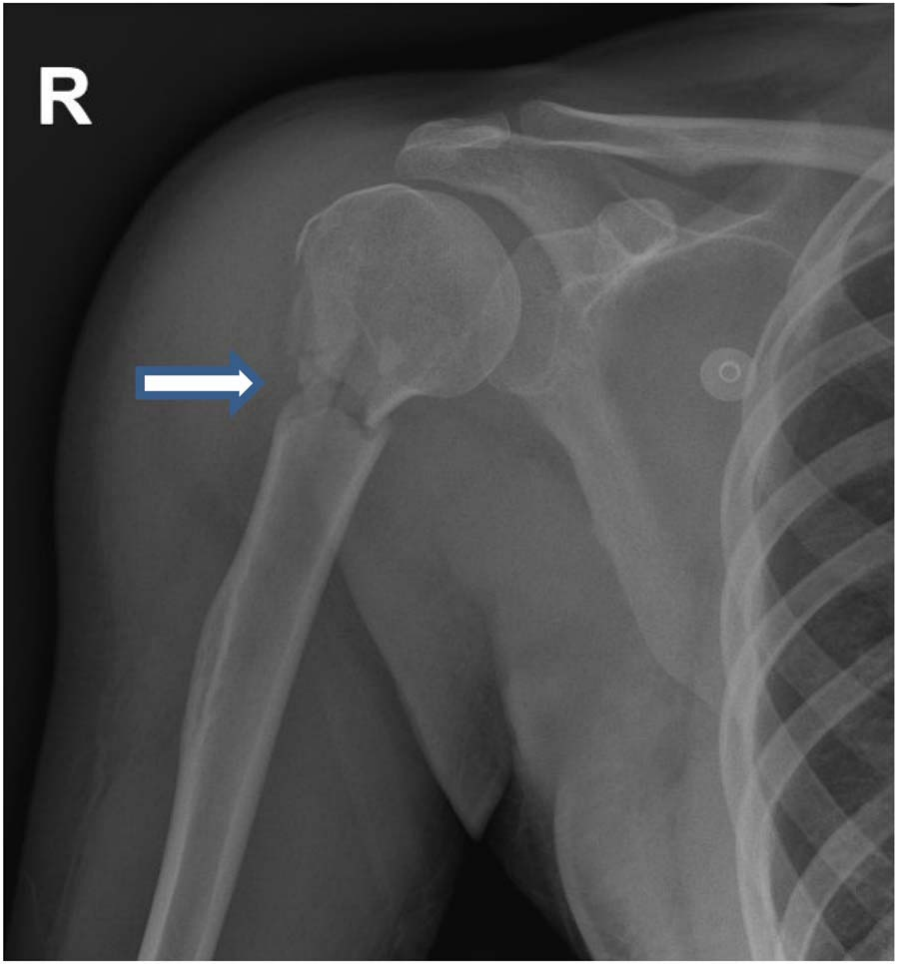
Upper limb X-ray revealed fracture of right Humerus.

In total, 11 units of RBC, 12 units of fresh frozen plasma and 12 units of platelet concentrate, 6 units cryoprecipitate, Prothrombin complex concentrate (1000 units), fibrinogen (12 gram) and tranexamic acid were transfused (ROTEM-guided). The patient also received oxytocin (40 units) and methergine (0.4 mg) injections for the prevention and control of postpartum hemorrhage and then transferred to the hybrid operating room for imaging. Computed tomography (CT) scan revealed small laceration in the right lobe of the liver (segment 8) and another laceration at the upper pole of the right kidney without active bleeding. However, there was a focus of contrast blush seen in the arterial phase at the right lateral side of the uterine wall, which increased in the venous and delayed phases suggesting active arterial bleeding (Fig. 1a–c). The contrast extravasation is also seen within the endometrial cavity (Fig. 1c). Interventional radiologist was consulted, and he advised for close observation because these findings could be related to immediate postpartum uterine changes. The patient was transferred to the Trauma Intensive Care Unit (TICU) under mechanical ventilation, where she was hemodynamically stable. The last Intra-operative lab work showed hemoglobin level of 10 g and base excess was improved from −16 to −9 mEq/l. For completion of secondary survey, right shoulder X-ray was done and revealed a fracture of the right humerus head (Fig. 2). Few hours after admission to the TICU, the patient had post partum hemorrhage as frequent changing of blood-soaked packs was required and the hemoglobin dropped from 10 to 7 g. Sweeping of the uterine cavity was done by the obstetrician and small pieces of membranes was removed and no placental tissue was felt. Oxytocin drip 20 units in 500 ml was given intravenously and misoprostol 600 mcg was given rectally.

**Figure 3:**
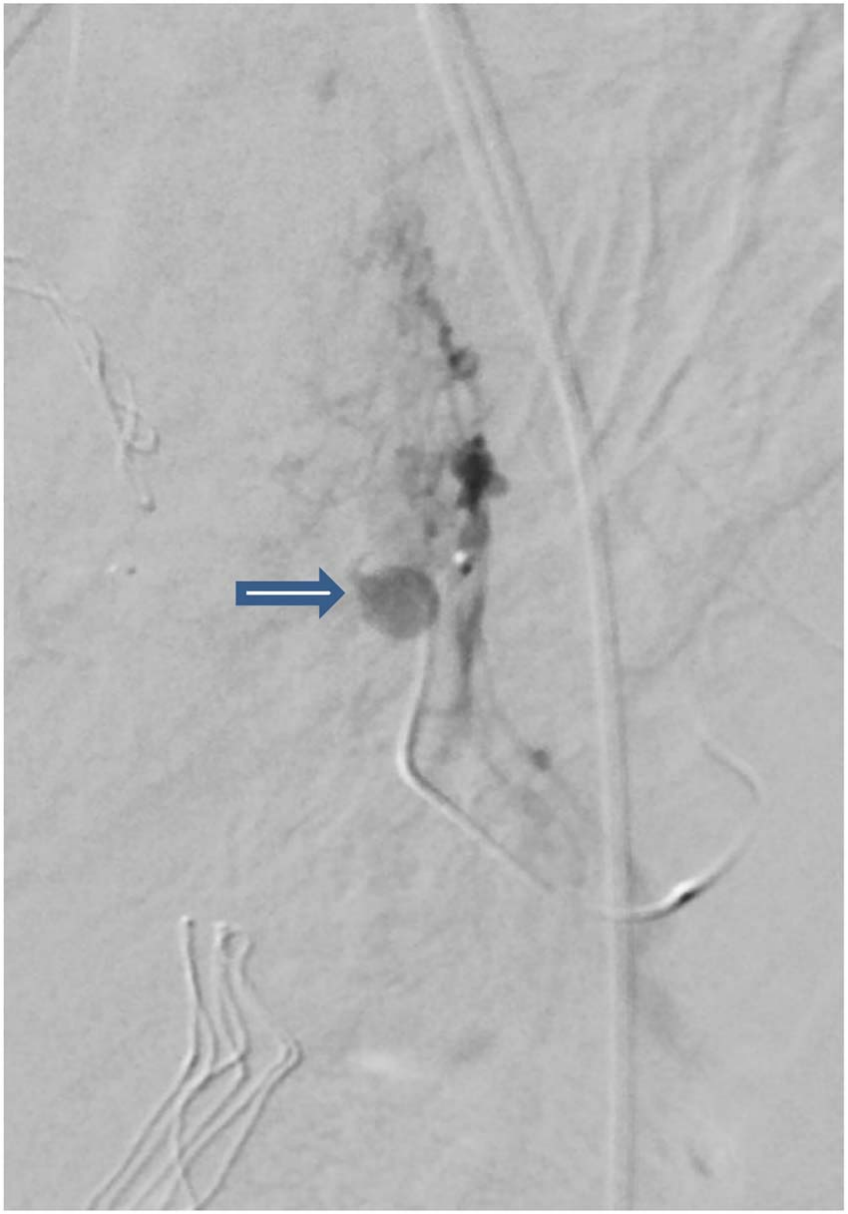
Pelvic angiogram revealed a pseudoaneurysm (see arrow) measuring ~7 mm arising from the branches of the left uterine artery.

**Figure 4:**
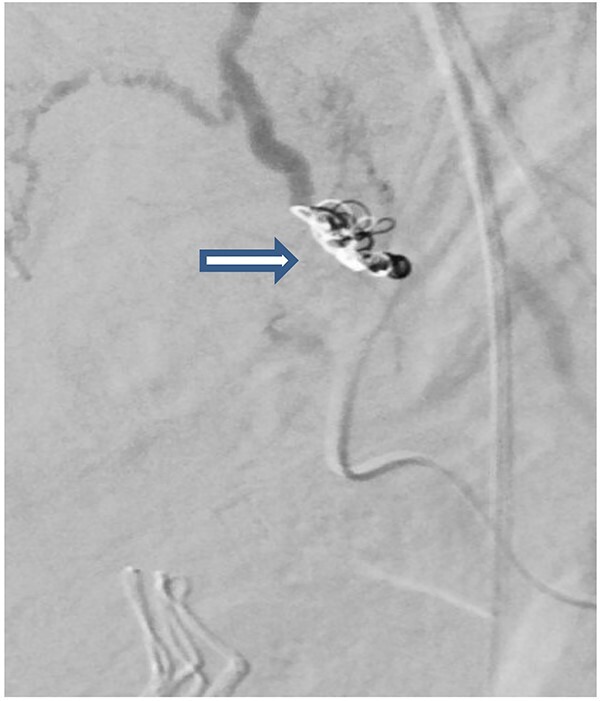
Post embolization angiogram showed satisfactory results after multiple Vortex coils were used to obliterate of feeding vessels.

The case was again discussed between trauma and obstetric teams for possible need for interventional radiology consultation. Angiogram showed tortuous arteries with parenchymal blush in the right side of uterine wall, which was related to postpartum status. Gelfoam embolization of the right uterine artery was done to reduce the flow. On the other side, angiogram detected a pseudoaneurysm measuring ~7 mm (Fig. 3). Multiple vortex coils were used to obliterate the feeding vessels and post embolization angiogram showed satisfactory results (Fig. 4).

On the second day, the patient had normal coagulation parameters, stable hemoglobin level and her acidosis resolved. She was taken back to the operating room where the packs were removed and no active bleeding was noted, the abdomen was closed, and the patient was transferred back to the TICU. She was extubated and had uneventful recovery. The patient was transferred to the trauma ward on the third hospital day. Her humerus fracture was evaluated by orthopedic team and treated conservatively.

During the hospital stay, the patient was seen by a trauma psychologist and received counseling for post-traumatic stress and to help grieve for the accident. She was discharged on the fifth postoperative day in a good condition and the abdominal clips were removed during the trauma out-patient clinic visit after 12 days.

**The infant female**: the infant was delivered with few non-effective breathings and was found to be covered with blood, had body blue color, bradycardic, and floppy with absent muscle tone. Her APGAR score was 2 at 1 min and then improved to 6 at 5 min. The neonate was resuscitated by the anesthesia team. Consequently, using positive pressure ventilation (PPV) for 30 s then intubated by size 2.5 endotracheal tube. When the NICU transport team arrived; endotracheal tube was changed to size 3.5 to suck large amount of blood from the airway and the stomach. Umbilical venous catheter was inserted, one bolus of saline 30 ml, and another one bolus of Dextrose 10% 6 ml was given.

The infant was passively cooled for 6 h then discontinued as the baby became neurologically well and cerebral function monitor showed normal traces. However, at age of 30 h, the baby developed single episode of tonic–clonic seizure, which was treated with phenobarbital. CT scan of the head was within normal.

**Figure 5:**
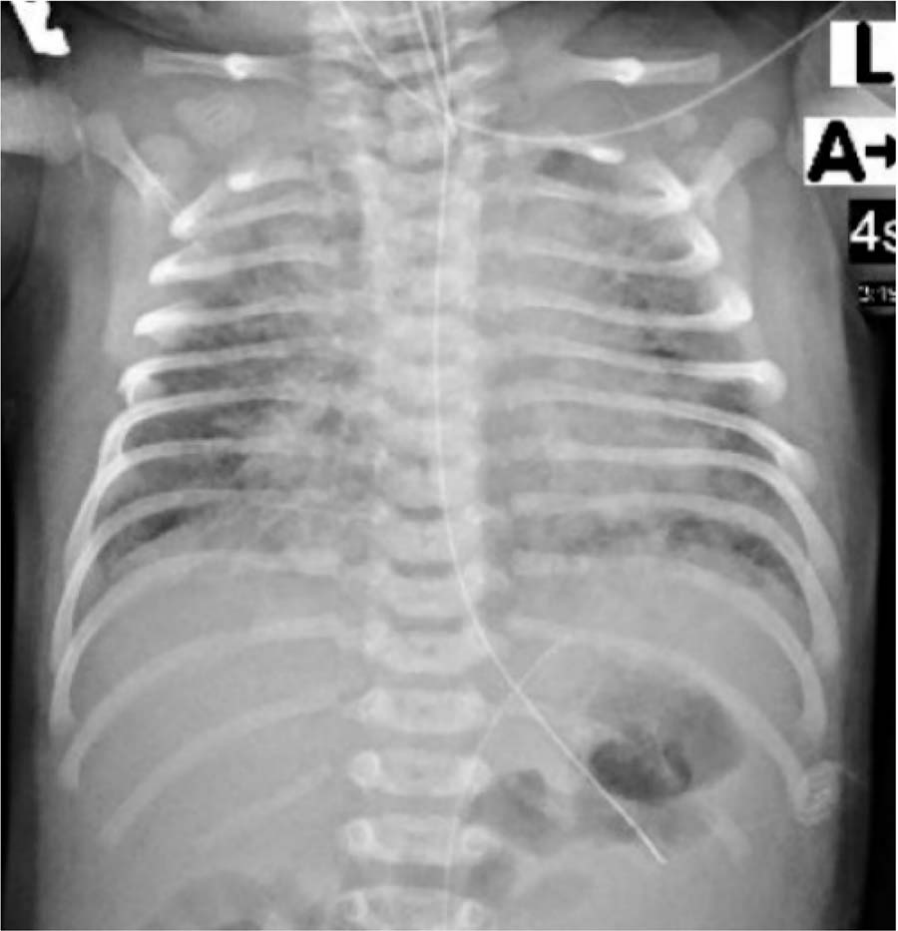
Chest X-ray of the neonate showed diffuse patchy pulmonary infiltrations.

The infant was kept on mechanical ventilation for 9 h and then required oxygen supplement by nasal cannula for 6 days and finally she was able to maintain oxygen saturation by self-breathing in room air. Her initial chest X-ray (Fig. 5) showed diffuse patchy pulmonary infiltrations, which was improved on the following days. The infant was fully recovered and discharged home after 7 days. The infant was seen in pediatric clinic and the last follow-up visit was after three months revealed her growth and development within the expected normal level.

## DISCUSSION

Blunt trauma in a full-term pregnancy may cause series of unique concerns that require a multi-disciplinary approach to ensure appropriate management. The present case showed a rare combination of retro-hepatic hemorrhage, placental abruption, and postpartum bleeding due a direct blunt trauma to the abdomen. The possibility of maternal and/or fetal injury is ~54% during the third trimester [5].

In this present case, the mother in her late pregnancy required care from different subspecialities including trauma surgery, obstetrics, anesthesia, ICU, orthopedics, NICU, interventional radiology and psychiatry. It is vital that these patients be identified early and if possible, brought to a level one trauma center where optimal integrated care can be provided.

There were challenges in this case; the unique physiologic change was hyperdynamic hypervolemic state, which made the assessment for shock challenging because the mother did not show signs of distress and collapse until delivery.

In addition to the typical hemorrhage sites of blunt trauma such as solid organ injury and retroperitoneal area, there were more potential sites of hemorrhage such as the uteroplacental and intrauterine hemorrhage.

Moreover, traumatic abruption of the placenta led to major maternal bleeding and consumption coagulopathy with thrombocytopenia, and hypofibrinogenemia.

Immediate control of surgical bleeding and attention to rotational thromboelastometry (ROTEM) finding to correct coagulopathy has led to a successful resuscitation.

Finally, postpartum hemorrhage is a serious condition that can cause significant morbidity and mortality. Early recognition and prompt treatment can lead to a full recovery.

Salome *et al*. documented that about 1 in 216 caesarean sections were complicated by significant morbidity from bleeding, and 1 in 14 females with severe morbidity progressed to maternal death. Furthermore, there were significant associations between abruptio placentae and atonic uterus which was most frequent cause of postpartum hemorrhage [6].

Medications and procedures used to control this kind of postpartum hemorrhage included intravenous oxytocin, ergometrine, misoprostol, prostaglandin F2- alpha, and finally uterine vessel ligation or embolization [7].

Another challenge was neonatal resuscitation in the operating room as the staff were not frequently resuscitating and managing the critically ill infant who had unplanned delivery in the operating room. Prior report showed that the death rate could be increased by 16% for every 30 s of delay in initiating ventilation for up to 6 min and increased by 6% for every 1 min of delay. Immediate and proper neonatal resuscitation procedures can reduce neonatal mortality by ~30% [8].

In conclusion, trauma in late pregnancy represents a significant clinical challenge for the trauma surgeon given the complexity of the pregnant woman with polytrauma. Maternal hemorrhage and coagulation abnormalities should be aggressively treated to optimize patient management. A proper organized multidisciplinary approach towards the patient and fetus in this situation necessitates expertise from multiple specialties, and the success of this interplay greatly affects the maternal and fetal outcomes.

## AUTHORS’ CONTRIBUTIONS

All authors have made a substantial contribution to the concept, design of the work and interpretation of data; and led the drafting and revising the manuscript critically for important intellectual content and approved the final version to be published.

## CONFLICT OF INTEREST STATEMENT

The authors declare that there is no conflict of interest.

## ACKNOWLEDGEMENT

Qatar National Library covered the open access publication fees.

## ETHICAL APPROVAL

The Medical Research Center of Hamad Medical Corporation has granted permission for this case report to be published on condition that no patient-identifiable data (including patient name and photograph) are included (IRB: MRC-04-22-521).
